# MARCH8 promotes the proteasomal degradation of foot-and-mouth disease virus VP1, VP2, and VP3 to negatively regulate viral replication

**DOI:** 10.1186/s13567-025-01521-z

**Published:** 2025-04-30

**Authors:** Mengge Yin, Xiangmin Li, Min Zhang, Qiongqiong Zhao, Haoyuan Wang, Huiyan Zhang, Zengjun Lu, Ping Qian

**Affiliations:** 1 National Key Laboratory of Agricultural Microbiology, Hubei Hongshan Laboratory, Huazhong Agricultural University, Wuhan, 430070 Hubei China; 2 College of Veterinary Medicine, Huazhong Agricultural University. Wuhan, Hubei, 430070 China; 3 Key Laboratory of Preventive Veterinary Medicine in Hubei Province, The Cooperative Innovation Center for Sustainable Pig Production, Wuhan, 430070 Hubei China; 4 Key Laboratory of Development of Veterinary Diagnostic Products, Ministry of Agriculture of the People’s Republic of China, Wuhan, 430070 Hubei China; 5 Hubei Jiangxia Laboratory, Wuhan, 430200 China; 6 State Key Laboratory for Animal Disease Control and Prevention, National Foot-and-Mouth Disease Reference Laboratory, Lanzhou Veterinary Research Institute, Chinese Academy of Agricultural Sciences, Lanzhou, 730046 Gansu China

**Keywords:** Membrane-associated RING-CH8 protein, foot-and-mouth disease virus, restriction factor, proteasomal degradation, K33-linked polyubiquitination

## Abstract

The host cell membrane-associated RING-CH8 protein (MARCH8) functions as an antiviral host factor by targeting viral envelope glycoproteins. Foot-and-mouth disease virus (FMDV) is a non-enveloped, positive-sense, single-stranded RNA virus. The potential impact of MARCH8 on FMDV replication remains uncertain. Here, we found that the overexpression of MARCH8 significantly inhibited FMDV replication in a dose-dependent manner. Conversely, the knockdown of MARCH8 facilitated FMDV replication. Specifically, MARCH8 interacted with VP1, VP2, and VP3, mediating their degradation in a proteasome-dependent manner. MARCH8 catalyzed the K33-linked polyubiquitination of VP1, VP2, and VP3. Moreover, the Lys210 residue of VP1, the Lys63 residue of VP2, and the Lys118 residue of VP3 were identified as critical target sites for MARCH8-mediated degradation. Overall, we conclude that MARCH8 is an intrinsic antiviral factor against FMDV.

**Supplementary Information:**

The online version contains supplementary material available at 10.1186/s13567-025-01521-z.

## Introduction

Membrane-associated RING-CH 8 (MARCH8), the first discovered mammalian MARCH protein, is a RING finger E3 ubiquitin ligase [1, 2]. Although MARCH8 has been shown to downregulate various host transmembrane proteins, such as MHC-II, CD44, CD81, and CD86 [3, 4], its physiological functions remain largely unclear. Recently, MARCH8 has emerged as an antiviral factor. MARCH8 inhibits human immunodeficiency virus type 1(HIV-1) infection by binding to and downregulating HIV-1 envelope glycoprotein (Env), whereas its tyrosine motif mutation (^222^Axx^L225^) cannot downregulate HIV-1 Env [5, 6]. Lun et al. reported that MARCH8 degrades Ebola virus glycoprotein (EboV GP) and the spike (S) protein of severe acute respiratory syndrome coronavirus 2 (SARS-CoV-2). The cytoplasmic tail of vesicular stomatitis virus G-glycoprotein (VSV G) plays an important role in MARCH8-mediated ubiquitination and degradation [7]. Similarly, the cytoplasmic tail of the murine leukemia virus (MLV) envelope is critical for MARCH8-mediated inhibition [8]. MARCH8 induces the ubiquitination and degradation of influenza virus (IAV) M2 in lysosomes, thereby suppressing IAV infection [9]. BST-2 recruits MARCH8 to recognize and degrade the PEDV N protein [10]. In addition, Kumar et al. reported that MARCH8 facilitates infection with hepatitis C virus (HCV), Zika virus (ZIKV), and dengue virus (DENV) [11].

Foot-and-mouth disease (FMD) is an acute, contagious, and economically significant viral disease that affects wild and domestic cloven-hoofed animals, including cattle, swine, sheep, goats, and buffaloes [12]. Foot-and-mouth disease virus (FMDV) belongs to the genus *Aphthovirus* within the *Picornaviridae* family [13]. Its genome is approximately 8.5 kb long and encodes a large polyprotein. Subsequently, the polyprotein is cleaved to generate 15 mature proteins and several precursors, mainly by the viral proteases L and 3C [14].

During FMDV infection, various host factors can regulate viral replication through multiple mechanisms. Dihydroorotase (CAD) facilitates FMDV replication via its ACTase domain [15]. Heat shock protein 60 (HSP60) plays a role in FMDV replication at entry stages [16]. Cellular beclin1 and vimentin potentiate FMDV replication by interacting with FMDV 2C [17]. DDX56 inhibits viral induction of IFN-β at the IRF3 level and cooperates with FMDV 3A to decrease IRF3 phosphorylation, thereby increasing FMDV replication [18]. In contrast, early growth response gene-1 (EGR1) enhances IFN production by promoting TBK1 phosphorylation to suppress FMDV replication [19]. Cyclophilin A (CypA) degrades FMDV L^pro^ and 3A via the proteasome pathway, and its PPIase activity is required for CypA to inhibit FMDV proliferation [20]. DDX60 inhibits FMDV replication by suppressing IRES-driven translation [21]. However, further investigation is needed to determine whether additional host proteins influence FMDV replication.

Ubiquitination is a post-translational modification process in which ubiquitin molecules, through the action of a cascade of specialized enzymes, selectively target proteins within the cell for specific modifications. This process involves three distinct enzymes: E1 ubiquitin-activating enzymes, E2 ubiquitin-conjugating enzymes, and E3 ubiquitin-ligating enzymes [22–24]. Protein ubiquitination plays essential roles in various cellular processes, including genome integrity, inflammatory signalling, the cell cycle, and cell death, and dampens pathogens [25]. Among the E3 ubiquitin ligases, MARCH8 has been shown to play a significant role in the antiviral response. It mediates the ubiquitination of several viral proteins, including IAV M2, EboV GP, and HCV NS2, thereby affecting viral proliferation. Interestingly, FMDV VP3 has also been reported to undergo ubiquitination [26]. Therefore, we designed this study to investigate the potential interactions between MARCH8 and FMDV.

In this study, we identified MARCH8 as a host antiviral effector that significantly suppressed FMDV replication. Our data demonstrated that MARCH8 interacts with the VP1, VP2, and VP3 proteins and reduces their protein levels through its E3 ubiquitin ligase activity. Importantly, the VP1_K210R_, VP2_K63R_, and VP3_K118R_ mutants were resistant to MARCH8-mediated degradation. Additionally, MARCH8 inhibited FMDV replication via its ZF and TM domains.

## Materials and methods

### Cells, viruses, and chemicals

Swine kidney-6 (SK6), human embryonic kidney 293 T (HEK293T), and baby hamster kidney (BHK-21) cells were maintained in Dulbecco’s modified essential medium ((DMEM); Invitrogen, USA) supplemented with 10% foetal bovine serum (FBS), 100 U/mL penicillin, and 10 μg/mL streptomycin sulfate (GLENVIEW, USA) at 37 °C with 5% CO_2_. BSR-T7 cells, which stably express T7 RNA polymerase, were grown in DMEM supplemented with 10% FBS, penicillin (100 U/mL), streptomycin (10 μg/mL), and 1 mg/mL G418.

The FMDV O/HN/CHA/93 strain was maintained at the National Foot-and-Mouth Disease Reference Laboratory, Lanzhou Veterinary Research Institute, Chinese Academy of Agricultural Sciences. All viral experiments were conducted in the Biosafety Level 3 Laboratory, Lanzhou Veterinary Research Institute, Chinese Academy of Agricultural Sciences.

The chemicals and reagents used in the study included MG132 (S1748-1mg), ZVAD-FMK (C1202), NH4Cl (ST2030-100g), dimethyl sulfoxide (DMSO, ST038), protein A + G agarose (P2012), and 4′,6′-diamidino-2-phenylindole (DAPI, C1006), all of which were obtained from Beyotime Biotechnology.

### Antibodies

The following primary antibodies were used in this study: rabbit anti-FLAG monoclonal antibody, rabbit anti-GST polyclonal antibody, rabbit anti-GFP polyclonal antibody, rabbit anti-HA monoclonal antibody, mouse anti-GST monoclonal antibody, and mouse anti-GFP monoclonal antibody (all obtained from Proteintech); mouse anti-FLAG monoclonal antibody and mouse anti-HA monoclonal antibody (Medical & Biological Laboratories); mouse anti-β-tubulin monoclonal antibody and mouse anti-GAPDH monoclonal antibody (ABclonal); rabbit anti-FMDV VP1 polyclonal antibody (prepared in our laboratory); and porcine-derived monoclonal antibodies against FMDV VP1, VP2, and VP3, as well as porcine anti-FMDV serum, which were kindly provided by Dr Zengjun Lu (Lanzhou Veterinary Research Institute).

### Plasmids

MARCH8 (GenBank accession no. XM_005671227.3) and its mutants were inserted into pCDNA3.1-Flag. UBI (GenBank accession no. MH035498.1) was inserted into PCAGGS-HA. Nine mutant plasmids of PCAGGS-HA-UBI (K6, K11, K27, K29, K33, K48, K63, KR, and K33R) were constructed using the Mut Express MultiS Fast Mutagenesis Kit V2 (Vazyme), as shown in Figure 4B. PCAGGS-HA-VP1, PCAGGS-HA-VP2, PCAGGS-HA-VP3, PCAGGS-HA-3C, PCAGGS-HA-3D, PEBG-GST-VP4, PEBG-GST-2B, PEBG-GST-3A, PEBG-GST-3B, PEBG-GST-VP1, PEBG-GST-VP2, PEBG-GST-VP3, and PEGFP-C1-2C were constructed and stored in our laboratory. Mutants of PEBG-GST-VP1, PEBG-GST-VP2, and PEBG-GST-VP3 were generated using the Mut Express MultiS Fast Mutagenesis Kit V2 (Vazyme).

### Plaque assay

BHK21 cells were cultured in 6-well plates and incubated with serially diluted virus samples for 2 h when the cells reached 80% confluency. Subsequently, 2% LMP (low melting point) agarose solution in 2 × DMEM containing 4% FBS and 2% penicillin‒streptomycin antibiotics was added to the PBS-washed cells. After 72 h, the cells were fixed with 10% (v/v) formaldehyde and then stained with 0.2% (m/v) crystal violet. The virus titres were calculated as plaque-forming units (PFU).

### Knockdown of MARCH8

MARCH8 knockdown was performed using short hairpin RNAs (shRNAs). Four shRNAs targeting MARCH8 were subsequently cloned and inserted into the pLKO.1 vector. HEK-293 T cells were co-transfected with psPAX2, pMD2. G, and the shRNA constructs for 48 h to generate lentiviruses. The lentivirus was then harvested and transduced into SK6 cells for subsequent experiments. The shRNA sequences were as follows: shRNA-1, sense, 5′- CCGGGCATCAGATCTCTGCCATTCCCTCGAGGGAATGGCAGAGATCTGATGCTTTTT-3′; antisense, 5′-AATTAAAAAGCATCAGATCTCTGCCATTCCCTCGAGGGAATGGCAGAGATCTGATGC; shRNA-2, sense, 5′-CCGGGGGACATTCCATGAGTCATTCCTCGAGGAATGACTCATGGAATGTCCCTTTTT-3′; antisense, 5′- AATTAAAAAGGGACATTCCATGAGTCATTCCTCGAGGAATGACTCATGGAATGTCCC -3′); shRNA-3, sense, 5′-CCGGGCAAAGTGTATGTACAATTATCTCGAGATAATTGTACATACACTTTGCTTTTT-3′; antisense, 5′-AATTAAAAAGCAAAGTGTATGTACAATTATCTCGAGATAATTGTACATACACTTTGC-3′; and shRNA-4, sense, 5′-CCGG GAAGAGACTCAAGGCTTATAACTCGAGTTATAAGCCTTGAGTCTCTTCTTTTT-3′; antisense, 5′-AATTAAAAAGAAGAGACTCAAGGCTTATAACTCGAGTTATAAGCCTTGAGTCTCTTC-3′.

### Quantitative reverse transcription‒PCR (RT‒qPCR)

Cell samples were collected, and total RNA was extracted using TRIzol reagent (Invitrogen, Grand Island, NY). Total RNA (1.0 μg) was reverse transcribed into cDNA using the HiScript® III RT SuperMix (Vazyme, China) according to the manufacturer’s instructions, followed by a SYBR Green PCR assay. The comparative cycle threshold (2^−ΔΔCT^) method was used to calculate changes in mRNA expression. The RT‒qPCR primers used were as follows: swine MARCH8, CATTTCTAAGGCTGGGGGTTC (sense) and GATTGGAGGGCGTGACAGAG (antisense); and GAPDH, ACATGGCCTCCAAGGAGTAAGA (sense) and GATCGAGTTGGGGCTGTGACT (antisense).

### Western blotting

The cell samples were harvested and lysed with lysis buffer (1.19% HEPES, 0.88% NaCl, 0.04% EDTA, 1% NP-40, and a protease inhibitor) on ice. The lysates were centrifuged at 12,000 RCF for 10 min at 4 °C and then boiled with 5 × loading buffer at 95 °C for 10 min. The proteins were separated by 12% SDS‒PAGE and transferred to PVDF (polyvinylidene fluoride) membranes. The membranes were blocked with 5% skim milk for 2 h, followed by washing with PBST (0.1% Tween-20 and PBS). The membranes were then incubated with primary antibodies at room temperature for 2 h, washed five times with PBST and incubated with the corresponding horseradish peroxidase (HRP)-conjugated secondary antibody at room temperature for 1 h. Finally, the protein bands were detected using enhanced chemiluminescence (ECL).

### Coimmunoprecipitation (co-IP)

HEK293T cells were seeded in twelve-well plates and co-transfected with the appropriate plasmids. After 24 h, the cells were collected and lysed with lysis buffer containing protease inhibitors. The lysates were then centrifuged at 12 000 RCF for 10 min at 4 °C to remove cellular debris. For each sample, the lysate was incubated with the indicated antibody on a rolling incubator at 4 °C for 4 h or overnight. Subsequently, 20 µL of protein A/G agarose beads were added, and the mixture was incubated at 4 °C for 5 h. The mixture was subsequently washed five times with 0.8 mL of lysis buffer. Immunocomplexes captured on protein A/G agarose beads were then subjected to western blot analysis.

### IFA (immunofluorescence assay)

HEK293T cells were seeded in 24-well plates and co-transfected with the indicated plasmids for 24 h. The cells were then fixed with an acetone/methanol mixture (1:1) at −20 °C for 10 min. Following fixation, the cells were blocked with 2% BSA in PBS for 1 h at 37 °C. The anti-HA and anti-Flag primary antibodies were incubated with the cells at 37 °C for 2 h. Subsequently, the cells were washed with PBS and incubated with fluorochrome-conjugated secondary antibodies at 37 °C for 1 h. The cells were then washed four times with PBS and incubated with DAPI for 10 min at 37 °C. After five additional washes, images were captured using a Nikon confocal microscope.

SK6 cells were seeded in 24-well plates and transfected with the indicated plasmids. After 24 h, the cells were infected with FMDV for 4 h. The cells were fixed at −20 °C for 10 min and then blocked with 2% BSA at 37 °C for 1 h. The primary antibodies were incubated at 37 °C for 1 h. Subsequently, the cells were washed with PBS and incubated with fluorochrome-conjugated secondary antibodies at 37 °C for 1 h. The cells were washed four times with PBS and then incubated with DAPI at 37 °C for 10 min. After five additional washes, images were captured using a Nikon confocal microscope.

### Statistical analysis

GraphPad Prism software version 8 was used for statistical analysis. In this study, all the data are expressed as the means ± SD. Two-tailed Student t tests were used to compare the different treatment groups. Statistical significance was assessed on the basis of the p value: *p* < 0.05 (*), *p* < 0.01 (**), *p* < 0.001 (***).

## Results

### MARCH8 negatively modulates FMDV replication

**Figure 1:**
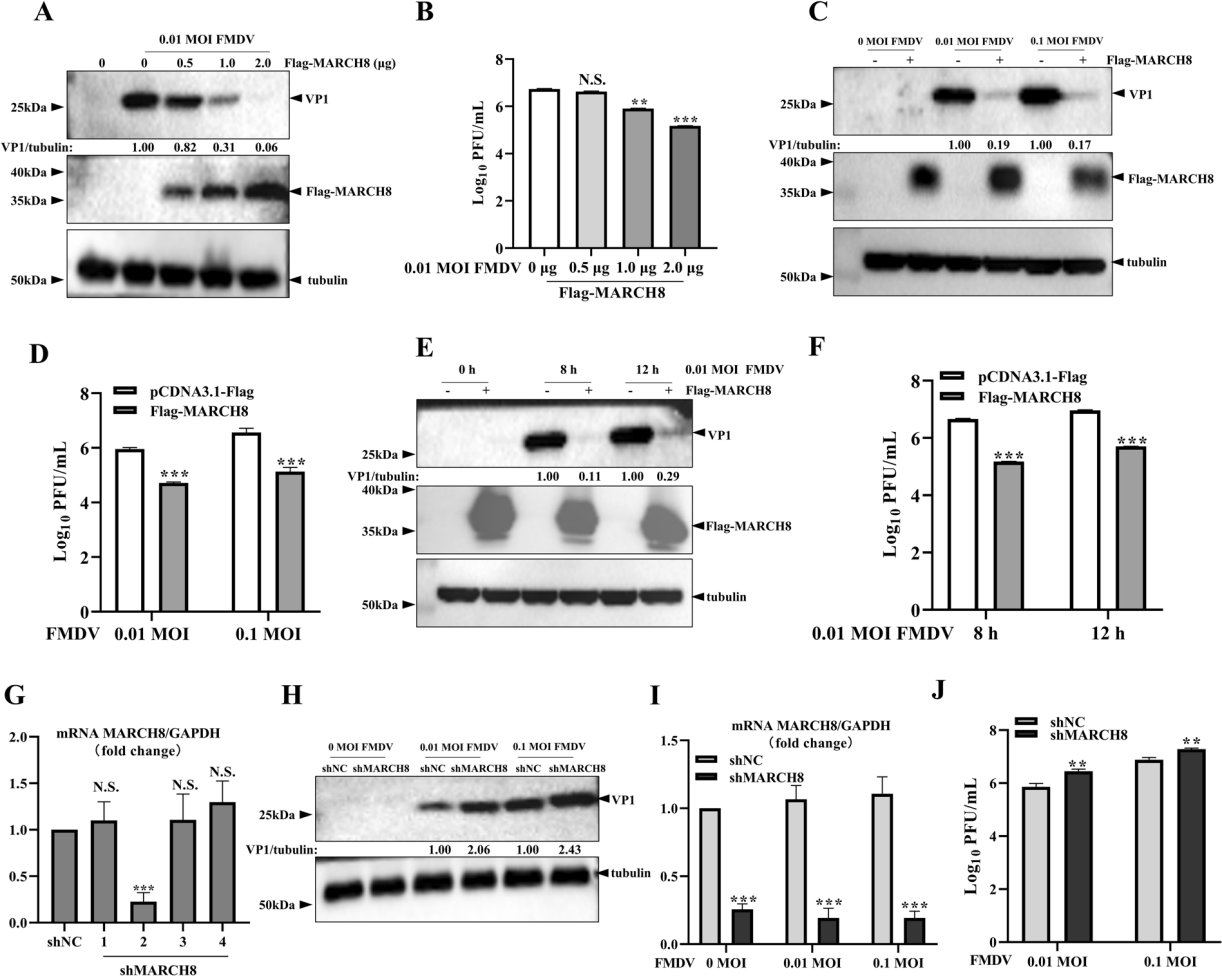
**MARCH8 overexpression inhibits FMDV propagation.** **A and B** SK6 cells in 6-well plates were transfected with increasing amounts of pCDNA3.1Flag-MARCH8 for 24 h, followed by infection with FMDV O/HN/CHA/93 (MOI = 0.01) for 8 h. VP1 protein levels were assessed by immunoblotting, and virus titres were detected by plaque assay. **C and D** SK6 cells in 6-well plates were transfected with 2 μg pCDNA3.1Flag or pCDNA3.1Flag-MARCH8 for 24 h and then infected with FMDV O/HN/CHA/93 (MOI = 0.01 or 0.1) for 8 h. VP1 expression was analysed by western blotting, and virus titres were measured by plaque assay. **E and F** SK6 cells in 6-well plates were transfected with 2 μg pCDNA3.1Flag or pCDNA3.1Flag-MARCH8 for 24 h and then infected with 0.01 MOI FMDV O/HN/CHA/93 for the indicated times. Viral replication was determined by western blotting and plaque assays. **G** SK6 cells in 6-well plates were transduced with shNC or shMARCH8 lentivirus for 48 h, and RNA was extracted from the cells for RT‒qPCR analysis. **H‒J** SK6 cells in 6-well plates were transduced with shNC or shMARCH8 lentivirus for 48 h and then infected with FMDV (MOI = 0.01 or 0.1) for 8 h. VP1 protein levels were analysed by western blotting, virus titres were determined by plaque assay, and endogenous MARCH8 expression was assessed by RT‒qPCR. The data are shown as the means ± SD. *, *P* < 0.05; **, *P* < 0.01; ***, *P* < 0.001; N.S.: not significant.

To investigate the impact of MARCH8 on FMDV replication, swine kidney-6 (SK6) cells overexpressing MARCH8 were infected with FMDV O/HN/CHA/93 at a multiplicity of infection (MOI) of 0.01 for 8 h. Western blotting results revealed that the ectopic expression of MARCH8 inhibited VP1 expression in a dose-dependent manner (Figure 1A). Similarly, the FMDV titres decreased 1.26-, 6.31- and 39.82-fold with increasing levels of MARCH8, respectively (Figure 1B). To further examine the effect of MARCH8 overexpression on FMDV replication at different MOIs, SK6 cells were transfected with the Flag-MARCH8 plasmid and then infected with increasing amounts of FMDV O/HN/CHA/93 (MOIs of 0.01 and 0.1) for 8 h. As shown in Figures 1C and D, MARCH8 overexpression resulted in 5.26- and 5.88-fold decreases in VP1 protein expression and 17.80- and 22.41-fold decreases in FMDV titres, indicating that MARCH8 suppresses FMDV replication at different MOIs. Additionally, SK6 cells were transfected with the Flag-MARCH8 plasmid and then infected with FMDV O/HN/CHA/93 (MOIs of 0.01) for the indicated time points. As shown in Figures 1E and F, VP1 protein expression was decreased 9.09- and 3.44-fold, and FMDV titres were decreased 9.09- and 3.44-fold at 8 and 12 h post-infection (hpi), respectively.

To confirm these results, endogenous MARCH8 was knocked down using short hairpin RNA (shRNA). As shown in Figure 1G, shMARCH8-2 resulted in a 4.17-fold decrease in the mRNA expression level of MARCH8, indicating that shMARCH8-2 effectively knocked down MARCH8-2. Therefore, this construct was used to assess the influence of endogenous MARCH8 on FMDV replication. The results of western blotting and plaque assays revealed that VP1 protein expression increased 2.06- and 1.43-fold, and FMDV titres increased 3.98- and 2.63-fold when MARCH8 was knocked down (Figures 1H–J). Collectively, these results demonstrate that MARCH8 inhibits FMDV replication.

### MARCH8 interacts with VP1, VP2, and VP3

**Figure 2:**
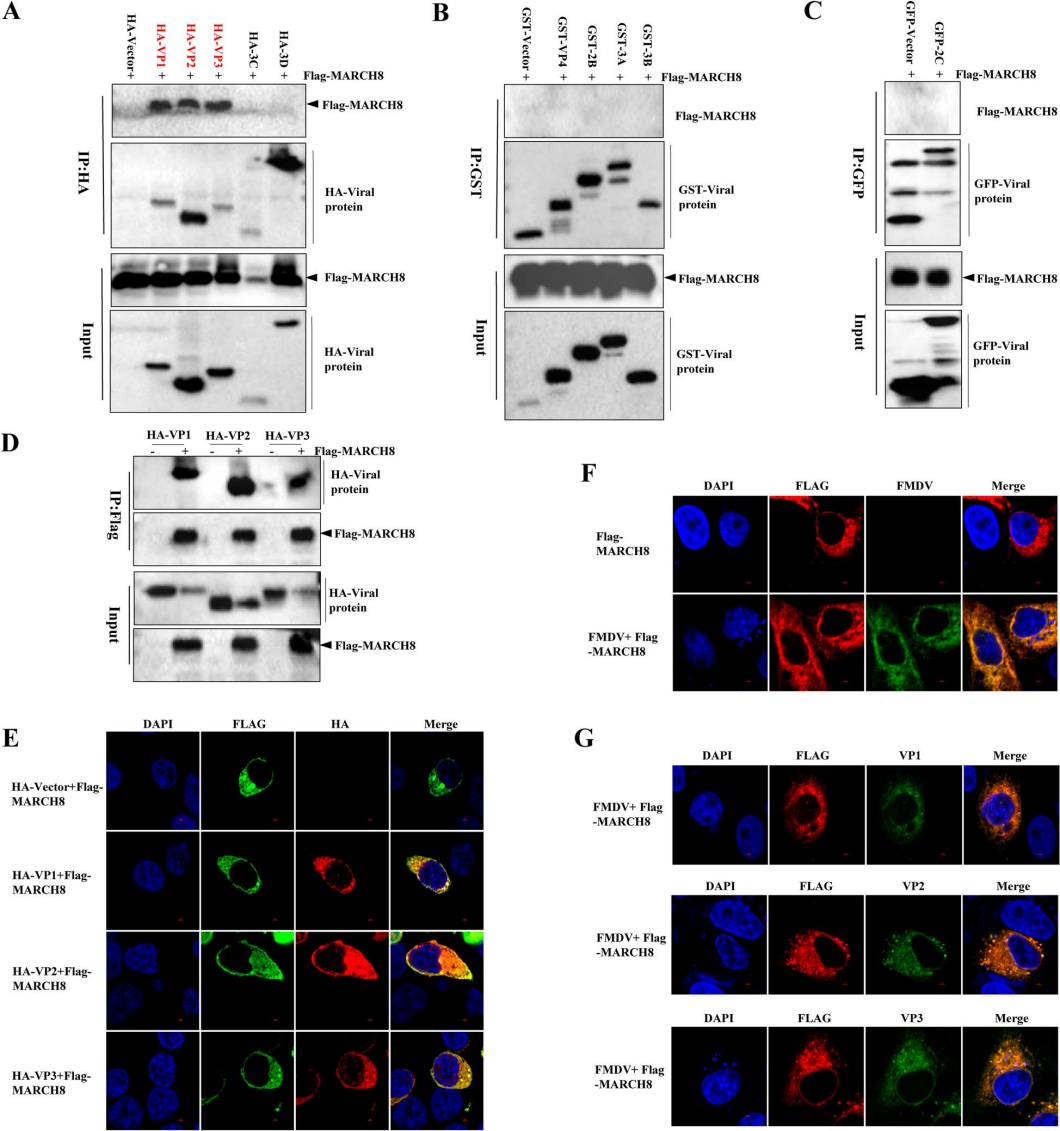
**MARCH8 interacts with VP1, VP2 and VP3.** **A** HEK293T cells were co-transfected with 1 μg PCAGGS-HA, HA-VP1, HA-VP2, HA-VP3, HA-3C, or HA-3D and 1 μg pCDNA3.1Flag-MARCH8 for 24 h. The resulting cellular lysates were incubated for co-IP analysis. **B** HEK293T cells were co-transfected with 1 μg of PEBG-GST, GST-VP4, GST-3A, GST-3B, or GST-2B and 1 μg of pCDNA3.1Flag-MARCH8 for 24 h. The resulting cellular lysates were incubated for co-IP analysis. **C** HEK293T cells were co-transfected with 1 μg of PEGFP-C1 or GFP-2C and 1 μg of pCDNA3.1Flag-MARCH8 for 24 h. The resulting cellular lysates were incubated for co-IP analysis. **D** HEK293T cells were co-transfected with 1 μg pCDNA3.1Flag or pCDNA3.1Flag-MARCH8 and 1 μg HA-VP1, HA-VP2, or HA-VP3 for 24 h. The resulting cellar lysates were incubated for co-IP analysis. **E** Confocal assays of HEK293T cells transfected with 0.4 μg PCAGGS-HA, HA-VP1, HA-VP2, or HA-VP3 and 0.2 μg pCDNA3.1Flag-MARCH8 for 24 h, followed by fixation and staining with anti-Flag (green), anti-HA (red) and DAPI nuclear stain (blue). Images were captured by confocal microscopy. **F and G** SK6 cells were transfected with 0.5 μg pCDNA3.1Flag or pCDNA3.1Flag-MARCH8 for 24 h and then subjected to FMDV O/HN/CHA/93 for 4 h. The cells were fixed and incubated with anti-FMDV serum, anti-VP1, anti-VP2, or anti-VP3 and anti-Flag antibodies, followed by incubation with secondary antibodies conjugated with TRITC (red) and FITC (green). Nuclei were stained with DAPI (blue), and images were captured by confocal microscopy.

On the basis of the above findings, we first performed co-IP to examine whether MARCH8 interacts with the structural and nonstructural proteins of FMDV. HEK293T cells were co-transfected with individual FMDV plasmids (VP1, VP2, VP3, VP4, 2B, 2C, 3A, 3B, 3C, and 3D) and the Flag-MARCH8 plasmid. Co-IP results indicated that MARCH8 could interact with the structural proteins VP1, VP2, and VP3 (Figures 2A–C). To further confirm these interactions, reverse immunoprecipitation was conducted by co-transfecting HEK293T cells with Flag-MARCH8 or empty vector plasmids and plasmids encoding HA-tagged VP1, VP2, and VP3. As shown in Figure 2D, VP1, VP2, and VP3 efficiently coimmunoprecipitated with MARCH8. Additionally, confocal microscopy analysis confirmed the cytoplasmic colocalization of MARCH8 with VP1, VP2, and VP3 (Figure 2E).

To further validate the interaction between MARCH8 and VP1, VP2, and VP3, SK6 cells overexpressing MARCH8 were infected with FMDV O/HN/CHA/93 for 4 h. Subsequently, porcine anti-FMDV serum and antibodies specific to VP1, VP2, and VP3 were used to detect structural proteins via confocal microscopy. Confocal microscopy revealed clear colocalization of MARCH8 and FMDV structural proteins in the cytoplasm during FMDV infection (Figure 2F). As shown in Figure 2G, MARCH8 was also observed to colocalize with VP1, VP2, and VP3 during authentic infection. Taken together, these findings suggest that MARCH8 interacts with the FMDV structural proteins VP1, VP2, and VP3.

### MARCH8 degrades the VP1, VP2, and VP3 proteins by the proteasome pathway

**Figure 3:**
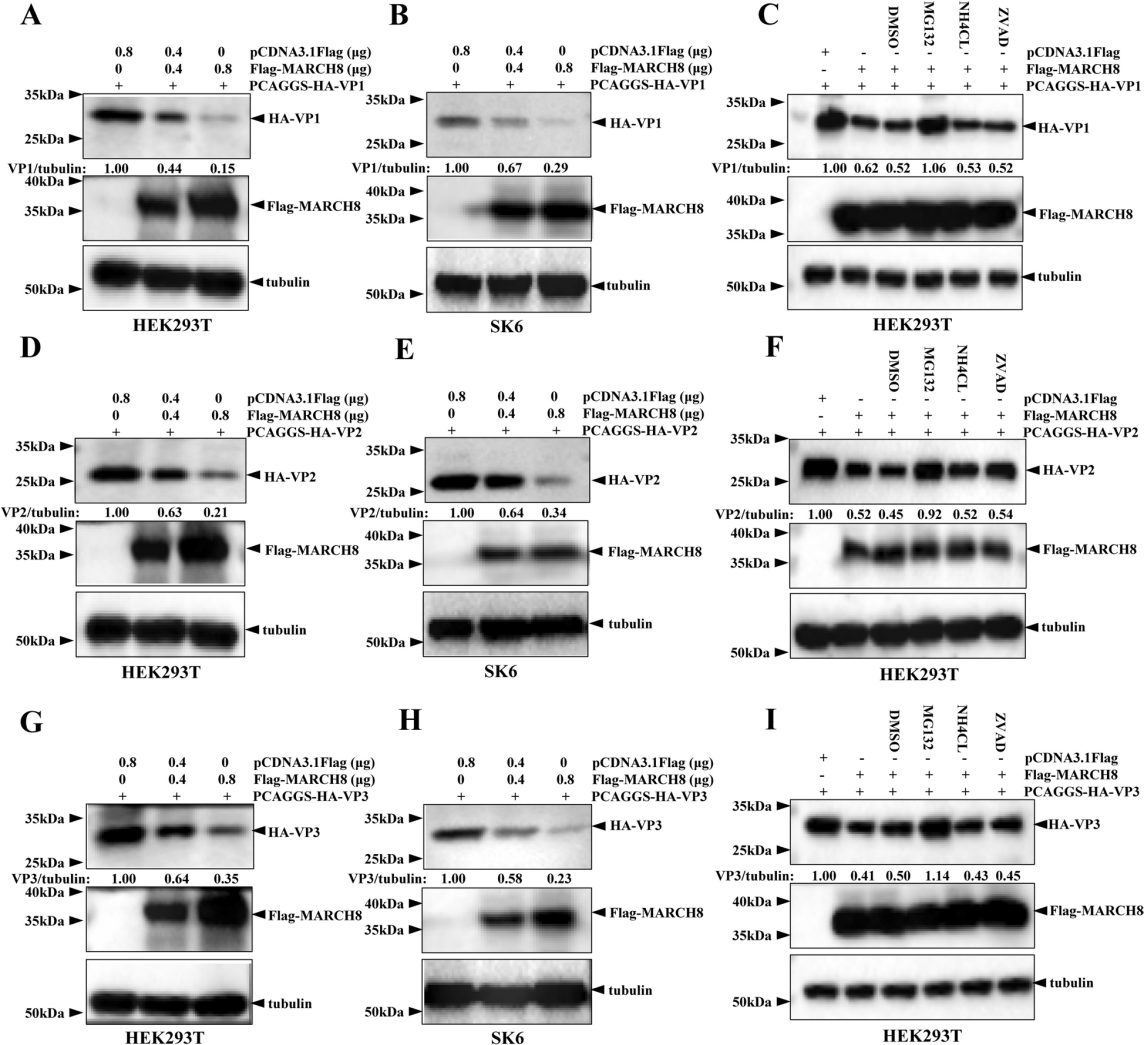
**MARCH8 degrades VP1, VP2, and VP3 via the proteasome pathway.** **A, D and G** HEK293T cells were co-transfected with 0.4 μg of HA-VP1, HA-VP2, or HA-VP3 and increasing amounts of pCDNA3.1Flag-MARCH8 for 24 h. The resulting cellular lysates were analysed by immunoblotting. **B, E and H** SK6 cells were co-transfected with 0.8 μg of HA-VP1, HA-VP2, or HA-VP3 and increasing amounts of pCDNA3.1Flag-MARCH8 for 24 h. The resulting cellular lysates were analysed by immunoblotting. **C, F and I** HEK293T cells were co-transfected with 0.4 μg pCDNA3.1Flag or Flag-MARCH8 and 0.4 μg HA-VP1, HA-VP2, or HA-VP3 for 12 h, followed by treatment with DMSO, NH4CL (10 mM), ZVAD (50 μM), or MG132 (10 μM) for 12 h. The resulting cellular lysates were analysed by immunoblotting.

Given that MARCH8 interacts with VP1, VP2, and VP3, we first examined whether MARCH8 affects the expression of these viral proteins. HEK293T cells were co-transfected with increasing doses of the Flag-MARCH8 plasmid and individual viral protein plasmids (HA-VP1, HA-VP2, and HA-VP3). As shown in Figures 3A, D and G, the protein levels of VP1, VP2, and VP3 gradually decreased with increasing MARCH8 expression. To investigate whether this effect also occurs in swine cells, SK6 cells were co-transfected with increasing amounts of the Flag-MARCH8 plasmid and plasmids encoding VP1, VP2, and HA-VP3. The results revealed that MARCH8 significantly reduced the protein expression of VP1, VP2, and VP3 in SK6 cells (Figures 3B, E and H). These results suggest that MARCH8 downregulates the protein levels of VP1, VP2, and VP3 in a dose-dependent manner.

Studies have shown that the proteasome and lysosome are major intracellular protein degradation systems in eukaryotic cells [27]. To determine whether MARCH8 degrades VP1, VP2, and VP3 through the proteasomal, lysosomal, or caspase pathways, HEK293T cells were co-transfected with Flag-MARCH8 plasmid and plasmids encoding VP1, VP2, and VP3 for 12 h and then treated with DMSO, the 10 μM proteasome inhibitor MG132, the 10 mM lysosome inhibitor NH4Cl, or the 50 μM pancaspase inhibitor ZVAD-FMK for another 12 h. As shown in Figures 3C, F and I, treatment with MG132 restored the protein levels of VP1, VP2, and VP3. In contrast, no significant changes in protein levels were observed with NH4Cl or ZVAD-FMK treatment compared with those with DMSO treatment. Collectively, our data indicate that MARCH8 mediates the degradation of VP1, VP2, and VP3 through the proteasome pathway.

### MARCH8 catalyzes K33-linked polyubiquitination of VP1, VP2, and VP3

**Figure 4:**
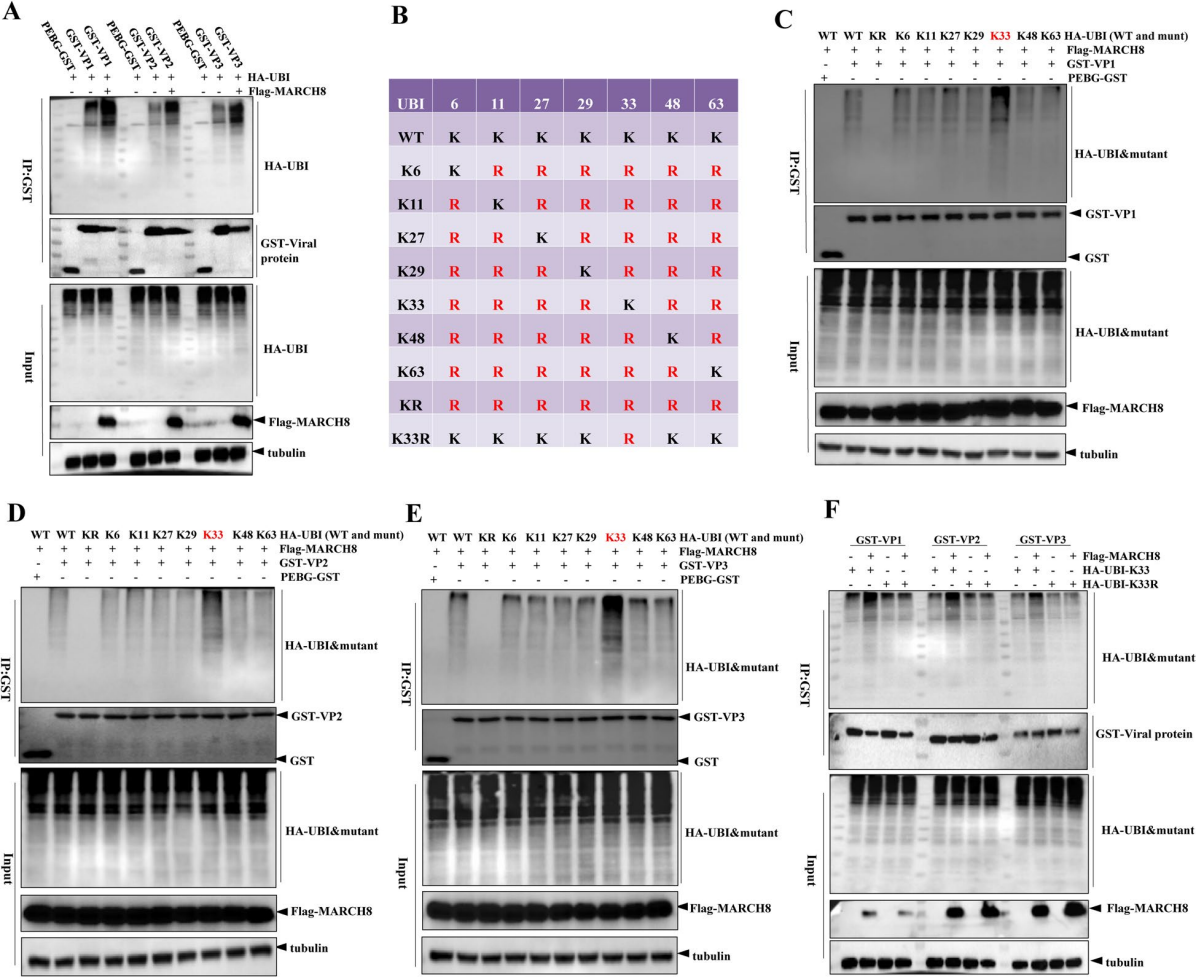
**MARCH8 induces the ubiquitination of VP1, VP2 and VP3.** **A** HEK293T cells were co-transfected with 1 μg pCDNA3.1Flag or pCDNA3.1Flag-MARCH8, 1 μg pCDNA3.0-HA-UBI or 1 μg PEBG-GST, GST-VP1, GST-VP2, or GST-VP3 for 24 h. The resulting cellular lysates were analysed by co-IP. **B** Schematic representation of ubiquitin (UBI) and nine UBI mutants (K6, K11, K27, K29, K33, K48, K63, KR and K33R). **C–E** HEK293T cells were co-transfected with 1 μg of pCDNA3.1Flag-MARCH8, 1 μg of pCDNA3.0-HA-UBI (wild type or mutants), and 1 μg of PEBG-GST, GST-VP1, GST-VP2, or GST-VP3 for 24 h. The resulting cellular lysates were analysed by co-IP. **F** HEK293T cells were co-transfected with 1 μg of pCDNA3.1Flag-MARCH8, 1 μg of pCDNA3.0-HA-UBI mutants (K33 and K33R), or 1 μg of PEBG-GST, GST-VP1, GST-VP2, or GST-VP3 for 24 h. The resulting cellular lysates were analysed by co-IP.

Ubiquitination, a key post-translational modification (PTM), plays an important role in combating viral infection by targeting pathogen proteins for degradation [28]. Previous studies have demonstrated that MARCH8 induces the degradation of IAV M2 at K78 through K63-linked polyubiquitination [9]. To explore whether MARCH8 mediates the degradation of VP1, VP2, and VP3 by ubiquitinating these proteins, HEK293T cells were co-transfected with the Flag-MARCH8 plasmid, HA-UBI plasmid and plasmids encoding GST-tagged VP1, VP2, and VP3. Co-IP results revealed that the ubiquitination of VP1, VP2, and VP3 was increased by MARCH8 overexpression (Figure 4A).

To further explore the specific type of polyubiquitin chains linked to VP1, VP2, and VP3 by MARCH8, the wild-type (WT) or eight mutant UBI plasmids (Figure 4B), the Flag-MARCH8 plasmid, and plasmids encoding GST-tagged VP1, VP2, and VP3 were co-transfected into HEK293T cells for 24 h. Co-IP analysis revealed that MARCH8-mediated ubiquitination levels of VP1, VP2, and VP3 were significantly increased when the cells were co-transfected with K33-ubiquitin (Figures 4C–E). To confirm the involvement of K33 in the polyubiquitination process, we replaced the lysine residue at position 33 of the UBI with an arginine (Figure 4B). As shown in Figure 4F, MARCH8 did not increase the ubiquitination levels of VP1, VP2, or VP3 in the presence of K33R-ubiquitin. These results indicate that MARCH8 specifically catalyzes the K33-linked polyubiquitination of VP1, VP2, and VP3.

### MARCH8 mediates the degradation of VP1, VP2, and VP3 at specific sites

**Figure 5:**
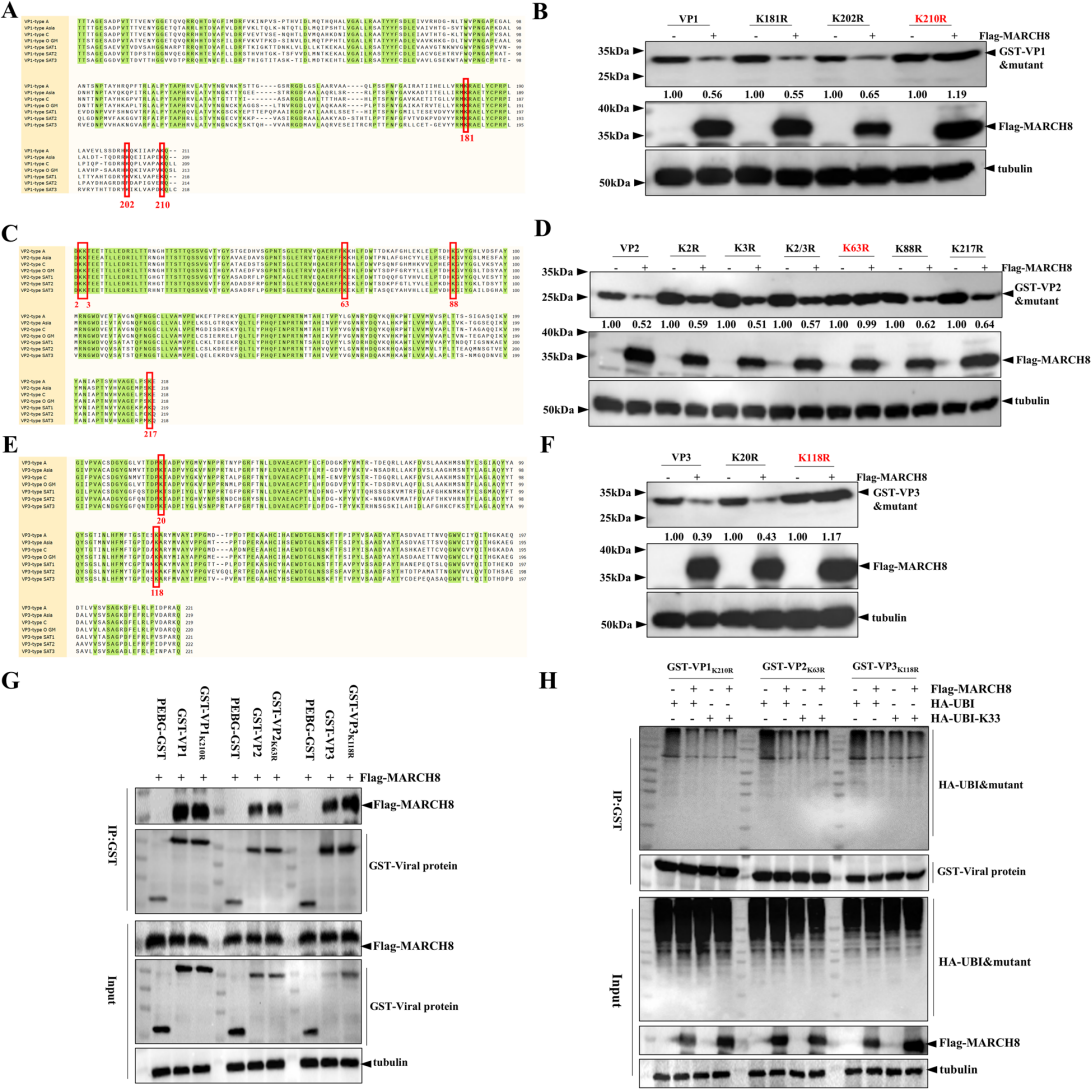
**MARCH8 mediates the degradation of VP1, VP2, and VP3 at specific sites. A, C and E** Sequence alignment of VP1, VP2, and VP3 from different FMDV serotypes (type-A: DQ533483.2; type-C: AM409325.1; type-Asia: MZ493234.1; type-SAT 1: MN275121.1; type-SAT 2: MZ090097.1; type-SAT 3:MG372727.1). **B, D and F** HEK293T cells were co-transfected with 0.4 μg pCDNA3.1Flag-MARCH8 and 0.4 μg wild-type or mutant VP1, VP2, or VP3 for 24 h. The resulting cellular lysates were analysed by immunoblotting. **G** HEK293T cells were co-transfected with 1 μg pCDNA3.1Flag-MARCH8 and 1 μg wild-type or mutant VP1, VP2, or VP3. After 24 h, the cellular lysates were analysed by co-IP. **H** HEK293T cells were co-transfected with 1 μg pCDNA3.1Flag-MARCH8, 1 μg pCDNA3.0-HA-UBI (wild type or mutant), and 1 μg GST-VP1_K210R_, GST-VP2_K63R_, or GST-VP3_K118R_ for 24 h. The cellular lysates were analysed by co-IP.

Protein ubiquitination can be classified into two types: canonical and nonlysine ubiquitination [29–31]. In canonical ubiquitination, the lysine residue of the target protein serves as the recognition site [22, 24]. For example, the Lys854 residue of infectious bursal disease virus (IBDV) VP3 is ubiquitinated by TRIM25 [32]. Similarly, K78 of influenza A virus (IAV) M2 is a key site for MARCH8-mediated degradation [9]. As shown in Figure 5A, we performed sequence analysis and identified three conserved lysine residues of FMDV VP1, namely, K181, K202, and K210. We then generated a panel of VP1 mutants to assess their expression levels in MARCH8-overexpressing cells. The results showed that the VP1_K210R_ substitution was not degraded by MARCH8, whereas the VP1_K181R_ and VP1_K202R_ mutants were still affected by MARCH8 (Figure 5B). Similarly, we identified five conserved lysine residues in VP2 (K2, K3, K63, K88 and K217) (Figure 5C). The western blotting results revealed that MARCH8 did not decrease the expression of VP2_K63R_ but did significantly affect the expression of VP2_K2R_, VP2_K3R_, VP2_K2/3R_, VP2_K88R_, and VP2_K217R_ (Figure 5D). Previous studies have shown that K20 and K118 of FMDV VP3 are conserved lysine residues [26]. We also performed sequence analysis of VP3. Therefore, we constructed K20R and K118R mutants to assess the effects of MARCH8 on their expression (Figure 5E). As shown in Figure 5F, the VP3_K118R_ mutant was resistant to MARCH8-induced degradation, indicating that MARCH8 degrades VP3 at K118. Taken together, these results suggest that MARCH8 mediates the degradation of VP1, VP2, and VP3 at specific sites.

To further determine whether the K210 residue of VP1, the K63 residue of VP2, and the K118 residue of VP3 are binding sites for MARCH8, co-IP experiments were performed in HEK293T cells co-transfected with these plasmids. The results showed that MARCH8 could bind to VP1_K210R_, VP2_K63R_, and VP3_K118R_ (Figure 5G), suggesting that the K210 residue of VP1, the K63 residue of VP2, and the K118 residue of VP3 are not involved in the associations between MARCH8 and VP1, VP2, or VP3. Furthermore, we investigated whether these sites are involved in the ubiquitination of VP1, VP2 and VP3 by MARCH8. As shown in Figure 5H, MARCH8-mediated ubiquitination of VP1_K210R_, VP2_K63R_, and VP3_K118R_ was decreased. However, MARCH8 did not enhance the K33-linked ubiquitination of the VP1_K210R_, VP2_K63R_ or VP3_K118R_ mutants. These results demonstrate that the K210 residue of VP1, the K63 residue of VP2, and the K118 residue of VP3 are the key ubiquitination sites of MARCH8.

To investigate the role of the K210 residue of VP1, the K63 residue of VP2, and the K118 residue of VP3 in the replication ability of FMDV, we generated recombinant wild-type FMDV O/HN/CHA/93 (rO/HN/93), rO/HN/93-K210R lacking the key degradation site of VP1, rO/HN/93-K63R lacking the key degradation site of VP2, rO/HN/93-K118R lacking the key degradation site of VP3, and rO/HN/93-KR lacking the key degradation sites of VP1, VP2 and VP3 using reverse genetics in BSR cells (Additional file 1A). The indirect immunofluorescence assay (IFA) results indicated that we successfully rescued rO/HN/93 and rO/HN/93-K210R but not rO/HN/93-K63R, rO/HN/93-K118R, or rO/HN/93-KR (Additional file 1B). To compare the replication abilities of wild-type FMDV (O/HN/93) and recombinant FMDV (rO/HN/93 and rO/HN/93-K210R), BHK21 cells were infected with the three viruses at an MOI of 0.01 and harvested at 4, 8, 12, 16, 20, and 24 hpi. The plaque assay results revealed no significant differences in the replication capacities of the three viruses (Additional files 1C and D). To determine whether MARCH8 inhibits rO/HN/93-K210R replication, SK6 cells overexpressing MARCH8 were infected with O/HN/93, rO/HN/93, or rO/HN/93-K210R. As shown in Additional file 1E and F, immunoblotting and plaque assay results revealed that MARCH8 reduced the VP1 protein levels and virus titres of the three viruses. These results demonstrate that destruction of the K210 site of VP1 does not affect the replication ability of FMDV, and rO/HN/93-K210R remains sensitive to MARCH8 inhibition.

### The W112 site of MARCH8 is critical for VP1, VP2, and VP3 degradation

**Figure 6:**
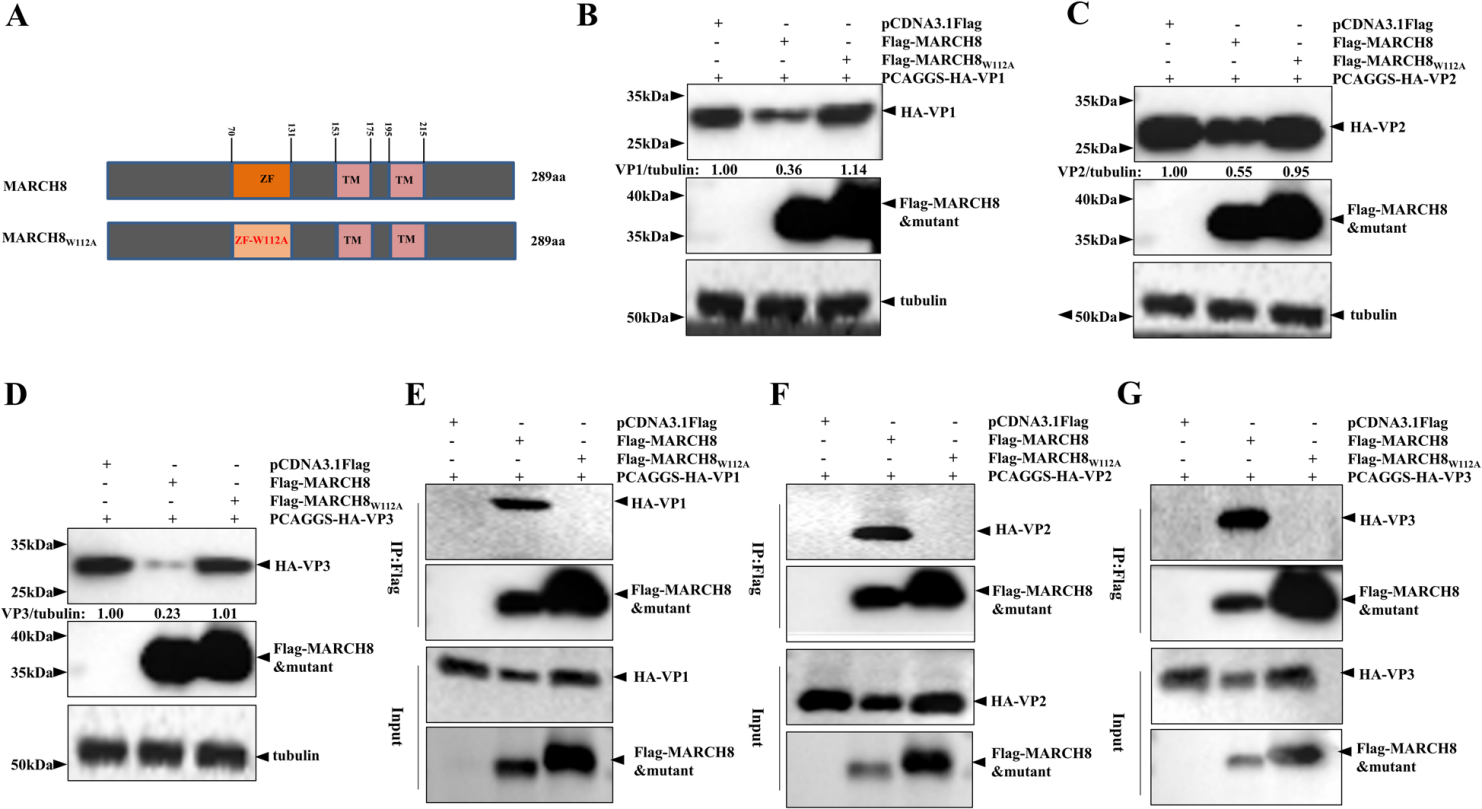
**MARCH8-induced degradation of VP1, VP2, and VP3 is dependent on its W112 site.** **A** Schematic diagram of MARCH8 and its mutant. **B–D** HEK293T cells were co-transfected with 0.4 μg pCDNA3.1Flag-MARCH8 or pCDNA3.1Flag-MARCH8_W112A_ and 0.4 μg HA-VP1, HA-VP2, or HA-VP3 for 24 h. The cellular lysates were analysed by immunoblotting. **E–G** HEK293T cells were co-transfected with 1.0 μg pCDNA3.1Flag-MARCH8 or pCDNA3.1Flag-MARCH8_W112A_ and 1.0 μg HA-VP1, HA-VP2, or HA-VP3 for 24 h. The resulting cellular lysates were analysed by co-IP.

MARCH8 contains a conserved RING-CH (ZF) domain for E3 ubiquitin ligase activity in the ubiquitination process [33]. We analysed the sequence of MARCH8 using InterPro [66] and constructed an inactive RING domain mutant (MARCH8_W112A_) to evaluate its effect on the expression of VP1, VP2, and VP3 (Figure 6A). The immunoblotting results revealed that sMARCH_W112A_ failed to reduce the protein expression of VP1, VP2, and VP3 (Figures 6B–D). We subsequently examined whether the W112 site of MARCH8 is essential for its interaction with VP1, VP2, and VP3. Co-IP assays revealed that only wild-type MARCH8 could interact with VP1, VP2, and VP3, whereas the catalytically inactive mutant MARCH8_W112A_ could not (Figures 6E‒G). Taken together, these results suggest that the E3 ubiquitin ligase activity of MARCH8 is essential for the degradation of the VP1, VP2, and VP3 proteins.

### sMARCH8 suppresses the replication of FMDV through its ZF and TM domains

**Figure 7:**
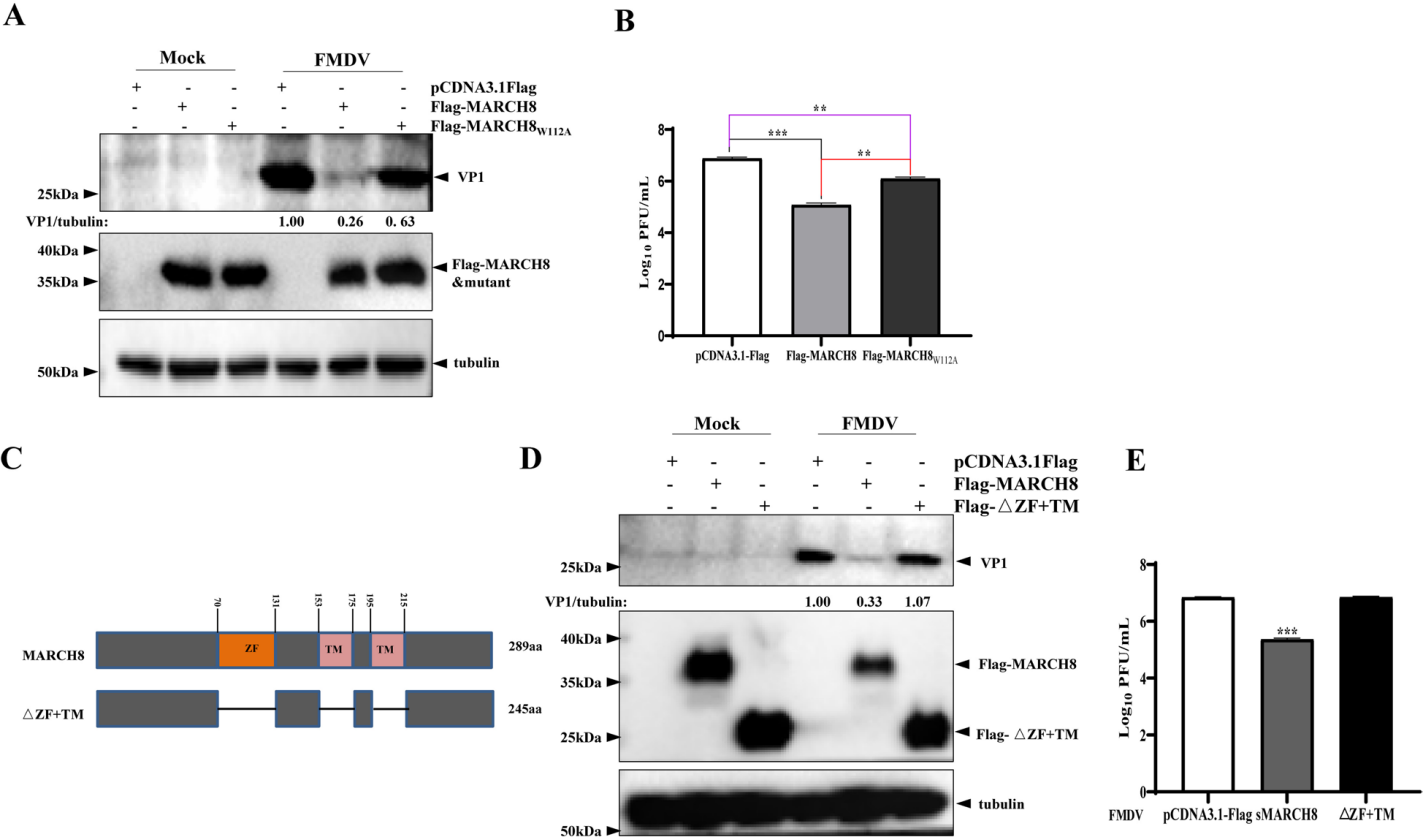
**MARCH8 inhibits FMDV replication by its ZF and TM domains.** **A and B** SK6 cells in 6-well plates were transfected with 2 μg pCDNA3.1Flag-MARCH8 or pCDNA3.1Flag-MARCH8_W112A_ for 24 h, followed by infection with 0.01 MOI FMDV O/HN/CHA/93 for 8 h. Viral replication was determined by western blotting and plaque assays.** C** Schematic representation of MARCH8 and its mutant. **D and E** SK6 cells in 6-well plates were transfected with 2 μg pCDNA3.1Flag-MARCH8 or pCDNA3.1Flag-△ZF + TM for 24 h, followed by infection with 0.01 MOI FMDV O/HN/CHA/93 for 8 h. Viral replication was determined by western blotting and plaque assays. The data are shown as the means ± SD. *, *P* < 0.05; **, *P* < 0.01; ***, *P* < 0.001.

Further experiments were performed to assess whether MARCH8 inhibits FMDV replication through its E3 ubiquitin ligase activity. The immunoblotting and plaque assay results revealed that, compared with the vector-expressing group, MARCH_8W112A_ suppressed FMDV replication, but the inhibitory effect decreased compared with that of the MARCH8-expressing group (Figures 7A and B), indicating that the E3 ubiquitin ligase activity of MARCH8 is partially involved in its antiviral activity against FMDV. In addition, MARCH8 contains two transmembrane (TM) domains [34, 35]. Therefore, we constructed an MARCH8 mutant (△ZF + TM) lacking the ZF and TM domains to assess its effect on FMDV replication (Figure 7C). As shown in Figures 7D and E, the MARCH8 mutant, which lacked the ZF and TM domains, was unable to inhibit FMDV replication. These findings indicate that the ZF and TM domains of MARCH8 are critical for its anti-FMDV activity.

## Discussion

FMD, caused by FMDV, is one of the most contagious diseases of livestock, with a significant impact on the quality and productivity of animal husbandry, resulting in enormous economic losses in the breeding industry [36, 37]. FMDV exhibits remarkable genetic diversity and antigenic variability [38]. Furthermore, after the acute phase of infection in cattle and sheep, a large proportion of animals can remain persistently infected for extended periods [39, 40]. This may lead to viral genetic reassortment, potentially generating new viral strains, which complicates efforts to control FMD. FMDV relies on host proteins to complete its life cycle, and its invasion is influenced by various host factors [41, 42]. Upon entry into the cell, FMDV is immediately detected by the host innate immune system, triggering multiple antiviral mechanisms that aim to inhibit viral replication and eliminate infection. However, through evolutionary adaptation, FMDV has developed strategies to subvert host defences, including proteolytic cleavage of host antiviral proteins, inhibition of interferon production and signalling, degradation of key immune signalling molecules, and hijacking of hijacked cellular proteins [43]. Therefore, understanding the interaction between FMDV and host proteins is crucial.

**Figure 8:**
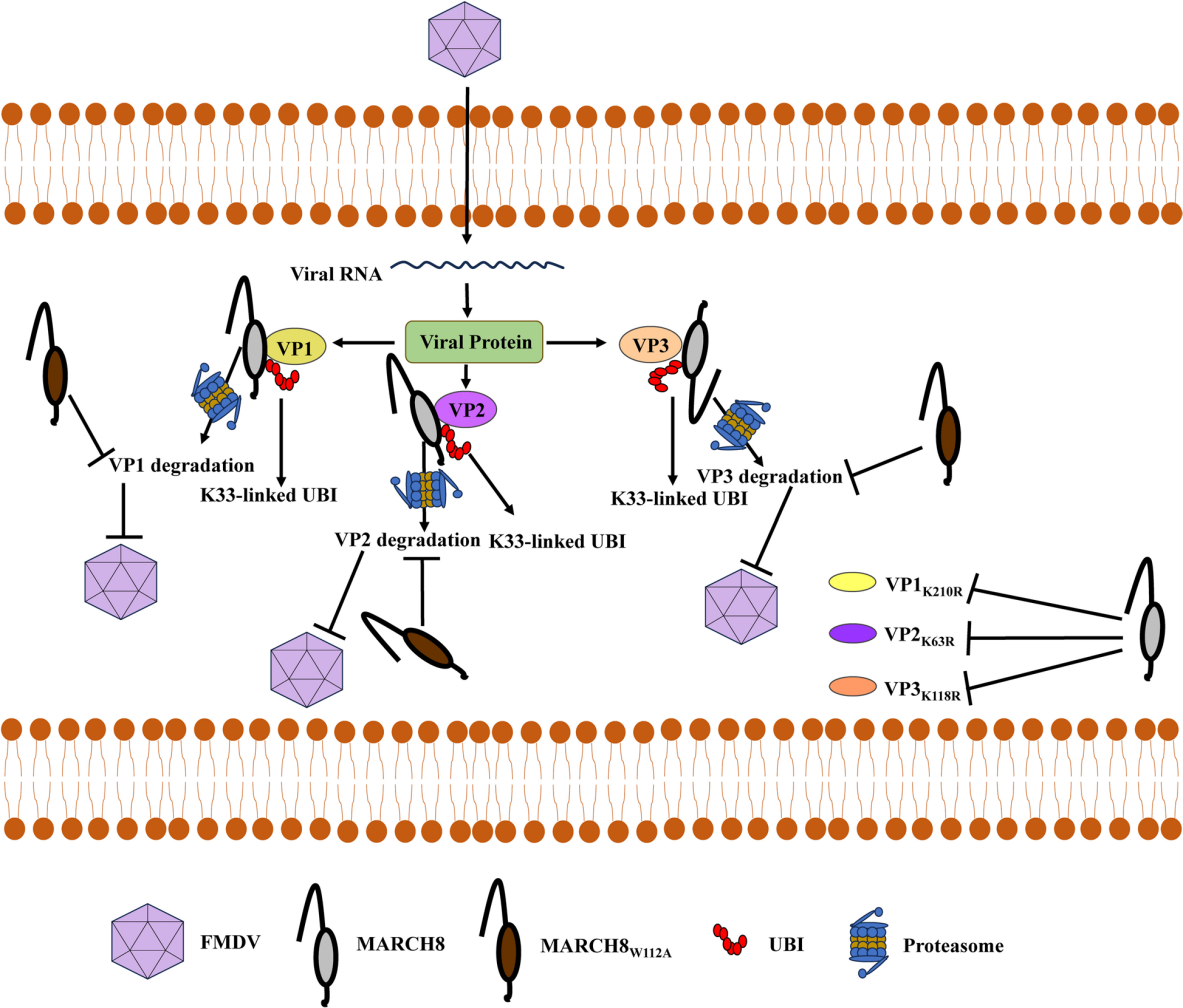
**Schematic model showing how MARCH8 negatively regulates FMDV infection.** MARCH8 inhibits FMDV replication by degrading the viral structural proteins VP1, VP2, and VP3 in a proteasome-dependent manner. The E3 ubiquitin ligase activity of MARCH8 is required for the degradation of VP1, VP2 and VP3. MARCH8 mediates K33-linked polyubiquitylation of VP1, VP2 and VP3. In addition, the VP1_K210R_, VP2_K63R_, and VP3_K118R_ substitutions cannot be degraded by MARCH8.

MARCH family proteins play important roles in many viral infections and exhibit both antiviral and proviral effects. For example, MARCH 1, 2 [44] and 8 [5] restrict HIV-1 infection; MARCH8 is a critical factor in the infection of several members of the *Flaviviridae* family, including HCV, DENV and ZIKV [11]. To date, MARCH8 is the most studied antiviral protein within the MARCH family. MARCH8 has been well characterized as a host restriction factor that restricts infection by various viruses [45]. However, its potential antiviral activity against picornaviruses, especially FMDV, remains to be fully investigated. In this study, MARCH8 targeted FMDV VP1, VP2, and VP3 for proteasomal degradation to suppress viral infection. In addition, the Lys210 residue of VP1, the Lys63 residue of VP2, and the Lys118 residue of VP3 are responsible for their degradation by MARCH8 (Fig. 8).

MARCH8 not only inhibits the replication of some viruses but is also occasionally hijacked by some viruses to promote their infection, suggesting that MARCH8 plays a dual role in host pathogen infection [34]. SVA 2AB binds LC3 or MAVS to degrade MARCH8, thereby inhibiting cell autophagy and type I IFN production to promote viral replication [46]. HCV NS2 hijacks MARCH8 to mediate its ubiquitination, thereby interacting with the endosomal sorting complex required for transport (ESCRT) machinery to facilitate viral envelopment [47]. Although some viruses can hijack MARCH8 to counterbalance its antiviral effect, MARCH8 can indeed interact with various viral proteins, influencing their expression and the viral life cycle. In our study, the ectopic expression of MARCH8 restricted FMDV replication, whereas the knockdown of endogenous MARCH8 increased FMDV replication. Further investigation revealed that MARCH8 interacted with the VP1, VP2, and VP3 proteins, mediating their degradation via the proteasomal pathway. Unfortunately, we were unable to detect endogenous MARCH8 expression despite several attempts using commercially available antibodies targeting human MARCH8. Additionally, our efforts to generate specific antibodies were unsuccessful.

Host proteins can mediate the ubiquitination of viral proteins, leading to their degradation and restriction of viral replication. For example, the tripartite motif-containing protein 32 (TRIM32) interacts with IAV polymerase basic protein 1 (PB1), mediating its K48-linked polyubiquitination and subsequent degradation, thereby limiting IAV infection [48]. Interferon-stimulated gene 12a (ISG12a) mediates K48-linked polyubiquitination of HCV NS5A at the K374 site to inhibit HCV infection [49]. Tripartite motif-containing protein 21 (TRIM21) promotes the ubiquitination-dependent degradation of H9N2 M1 at the R95 and K242 residues, thereby reducing IAV replication [50]. Endoplasmic reticulum (ER)-resident N-acetyltransferase 9 (Nat9) interacts with porcine reproductive and respiratory syndrome virus (PRRSV) glycoprotein 5 (GP5) and targets GP5 for K27-linked polyubiquitination and proteasomal degradation, suppressing viral infection [51]. Ring finger protein 2 (RNF2) facilitates the K48-linked polyubiquitination of porcine circovirus type 3 (PCV3) Cap to limit PCV3 replication [52]. In our study, we demonstrated that MARCH8 markedly increased the ubiquitination of VP1, VP2, and VP3 (Figure 4A), and further experiments revealed that MARCH8 catalyzed K33-linked polyubiquitination of these proteins.

The VP1, VP2, VP3 and VP4 proteins make up the FMDV capsid, with VP1, VP2, and VP3 located on the capsid surface [37, 53]. The VP1 protein is involved in viral adhesion, invasion, and the induction of protective immunity [54–56]. Compared with VP1 and VP3, VP2 is more conserved [57] and contains surface epitopes that induce humoral responses. Consequently, VP2 is useful for studying the evolutionary divergence of FMDV and for the development of FMDV detection tools [58, 59]. VP2 and VP3 are also important for capsid stability [57, 60, 61]. The VP3 protein contains important conformational neutralizing epitopes and receptor recognition sites of FMDV, which play critical roles in the innate immune response [57, 60, 62–64]. In the present study, the Lys210 residue of VP1, the Lys63 residue of VP2, and the Lys118 residue of VP3 were the key sites for MARCH8-mediated degradation. Therefore, we constructed four mutants of FMDV infectious clones (Additional file 1A). The rO/HN/93-K210R mutation was successfully rescued, whereas the rO/HN/93-K63R, rO/HN/93-K118R, and rO/HN/93-KR mutations could not be rescued. Notably, there was no difference in replication ability between rO/HN/93-K210R and rO/HN/93. More importantly, MARCH8 inhibited the replication of rO/HN/93-K210R, indicating that mutant viruses with a single-site mutation are not resistant to MARCH8 inhibition. Site analysis revealed that the Lys63 residue of VP2 and the Lys118 residue of VP3 were conserved in seven serotypes of FMDV (Additional files 1C and E), whereas the Lys210 residue of VP1 was not completely conserved in seven serotypes of FMDV (Additional file 1A). Thus, we hypothesize that the Lys63 residue of VP2 and the Lys118 residue of VP3 are critical for FMDV rescue and replication. Therefore, further research is needed to elucidate the role of these sites in the rescue and replication of FMDV.

The RING-CH domain of MARCH8 is widely recognized for its E3 ubiquitin ligase activity, which is crucial to its function [65]. In this study, we found that MARCH8 could not degrade or bind to VP1, VP2, or VP3 when its E3 ubiquitin ligase activity was lost. However, further investigation revealed that MARCH8_W112A_ still partially inhibited FMDV replication, suggesting that other domains of MARCH8 may also contribute to its ability to regulate FMDV proliferation. Furthermore, our data indicated that the ZF and TM domains of MARCH8 play crucial roles in its antiviral activity against FMDV. This study extends the antiviral spectrum of MARCH8.

This study first evaluated the effect of MARCH8 on FMDV replication. We found that MARCH8 significantly inhibited FMDV replication. Mechanistically, MARCH8 mediated K33-linked polyubiquitination and proteasomal degradation of VP1, VP2, and VP3. In addition, the ZF and TM domains of MARCH8 are utilized to inhibit FMDV replication. Collectively, our findings elucidate the antiviral mechanism of MARCH8 and provide potential strategies for controlling FMDV infection.

## Supplementary Information

**Additional file 1. K210R mutant FMDV is not resistant to MARCH8 inhibition. **(A) Schematic representation of an FMDV infectious clone. (B) BHK21 cells were infected with rO/HN/93, rO/HN/93-K210R, rO/HN/93-K63R, rO/HN/93-K118R, rO/HN/93-KR, or O/HN/9 for 8 h. The cells were fixed and incubated with primary anti-FMDV serum, followed by staining with FITC-conjugated secondary antibodies (green). Nuclei were stained with DAPI (blue). The fluorescence was observed using a fluorescence inverted microscope. (C and D) BHK21 cells were infected with 0.01 MOI O/HN/93, rO/HN/93, or rO/HN/93-K210R for the indicated time points. The virus titres were determined by a plaque assay. (E and F) SK6 cells in 6-well plates were transfected with 2 μg pCDNA3.1Flag or pCDNA3.1Flag-MARCH8 for 24 h, followed by infection with 0.01 MOI O/HN/93, rO/HN/93, or rO/HN/93-K210R for 8 h. Viral replication was determined by western blotting and plaque assays. The data are shown as the means ± SD. *, *P* < 0.05; **, *P* < 0.01; ***, *P* < 0.001.

## Data Availability

The original contributions presented in the study are included in the article, and further inquiries can be directed to the corresponding author(s).

## Abbreviations

MARCH8: membrane-associated RING-CH8 protein
FMDV: foot-and-mouth disease virus
K: lysine
R: arginine
W: tryptophan
SK6: swine kidney-6
HEK293T: human embryonic kidney 293 T
BHK-21: baby hamster kidney
DMSO: dimethyl sulfoxide
DAPI: 4′,6′-diamidino-2-phenylindole
RT‒qPCR: quantitative reverse transcription‒PCR
co-IP: coimmunoprecipitation
IFA: immunofluorescence assay
MOI: multiplicity of infection
UBI: ubiquitin
ZF: RING-CH
TM: transmembrane
PFU: plaque-forming units
