# Supplementary figures and images for: MARCH8 promotes the proteasomal degradation of foot-and-mouth disease virus VP1, VP2, and VP3 to negatively regulate viral replication
Source: Vet Res. 2025 Apr 30;56:96. doi: 10.1186/s13567-025-01521-z (PMC12044826; doi:10.1186/s13567-025-01521-z)

**
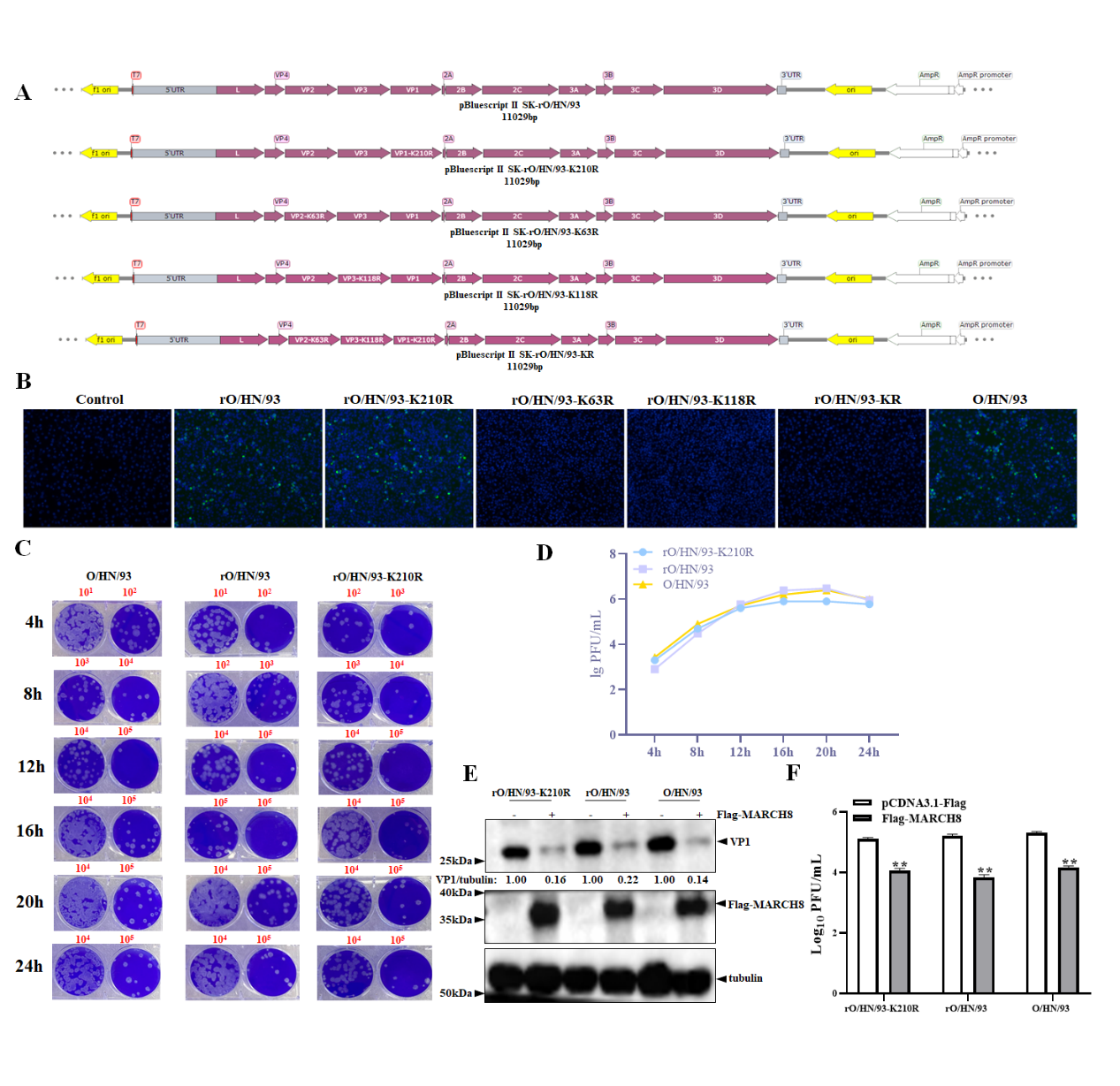
**

Supplement: Supplementary file 1 — Additional file 1. K210R mutant FMDV is not resistant to MARCH8 inhibition. (A) Schematic representation of an FMDV infectious clone. (B) BHK21 cells were infected with rO/HN/93, rO/HN/93-K210R, rO/HN/93-K63R, rO/HN/93-K118R, rO/HN/93-KR, or O/HN/9 for 8 h. The cells were fixed and incubated with primary anti-FMDV serum, followed by staining with FITC-conjugated secondary antibodies (green). Nuclei were stained with DAPI (blue). The fluorescence was observed using a fluorescence inverted microscope. (C and D) BHK21 cells were infected with 0.01 MOI O/HN/93, rO/HN/93, or rO/HN/93-K210R for the indicated time points. The virus titres were determined by a plaque assay. (E and F) SK6 cells in 6-well plates were transfected with 2 μg pCDNA3.1Flag or pCDNA3.1Flag-MARCH8 for 24 h, followed by infection with 0.01 MOI O/HN/93, rO/HN/93, or rO/HN/93-K210R for 8 h. Viral replication was determined by western blotting and plaque assays. The data are shown as the means ± SD. *, P < 0.05; **, P < 0.01; ***, P < 0.001. [file 13567_2025_1521_MOESM1_ESM.docx]
